# Development of phenotyping algorithms for hypertensive disorders of pregnancy (HDP) and their application in more than 22,000 pregnant women

**DOI:** 10.1038/s41598-024-55914-9

**Published:** 2024-03-15

**Authors:** Satoshi Mizuno, Maiko Wagata, Satoshi Nagaie, Mami Ishikuro, Taku Obara, Gen Tamiya, Shinichi Kuriyama, Hiroshi Tanaka, Nobuo Yaegashi, Masayuki Yamamoto, Junichi Sugawara, Soichi Ogishima

**Affiliations:** 1 Department of Informatics for Genomic Medicine, Tohoku Medical Megabank Organization, Tohoku University, 2-1, Seiryo-Machi, Aoba-Ku, Sendai, Miyagi 980-8575 Japan; 2 Department of Feto-Maternal Medical Science, Tohoku Medical Megabank Organization, Tohoku University, Miyagi, Japan; 3 Department of Molecular Epidemiology, Tohoku Medical Megabank Organization, Tohoku University, Miyagi, Japan; 4 Department of Statistical Genetics and Genomics, Tohoku Medical Megabank Organization, Tohoku University, Miyagi, Japan; 5 Tokyo Medical and Dental University, Tokyo, Japan; 6 Department of Gynecology and Obstetrics, Tohoku University Graduate School of Medicine, Tohoku University, Miyagi, Japan; 7 Department of Biochemistry and Molecular Biology, Tohoku Medical Megabank Organization, Tohoku University, Miyagi, Japan; 8 Suzuki Memorial Hospital, 3-5-5, Satonomori, Iwanumashi, Miyagi, Japan; 9 Advanced Research Center for Innovations in Next-Generation Medicine, Tohoku University, Miyagi, Japan

**Keywords:** Diseases, Medical research, Data mining, Computational biology and bioinformatics, High-throughput screening

## Abstract

Recently, many phenotyping algorithms for high-throughput cohort identification have been developed. Prospective genome cohort studies are critical resources for precision medicine, but there are many hurdles in the precise cohort identification. Consequently, it is important to develop phenotyping algorithms for cohort data collection. Hypertensive disorders of pregnancy (HDP) is a leading cause of maternal morbidity and mortality. In this study, we developed, applied, and validated rule-based phenotyping algorithms of HDP. Two phenotyping algorithms, algorithms 1 and 2, were developed according to American and Japanese guidelines, and applied into 22,452 pregnant women in the Birth and Three-Generation Cohort Study of the Tohoku Medical Megabank project. To precise cohort identification, we analyzed both structured data (e.g., laboratory and physiological tests) and unstructured clinical notes. The identified subtypes of HDP were validated against reference standards. Algorithms 1 and 2 identified 7.93% and 8.08% of the subjects as having HDP, respectively, along with their HDP subtypes. Our algorithms were high performing with high positive predictive values (0.96 and 0.90 for algorithms 1 and 2, respectively). Overcoming the hurdle of precise cohort identification from large-scale cohort data collection, we achieved both developed and implemented phenotyping algorithms, and precisely identified HDP patients and their subtypes from large-scale cohort data collection.

## Introduction

In recent years, many phenotyping algorithms have been developed for scalable and high-throughput cohort identification from electronic health records (EHRs)^1–5^ for genome- and phenome-wide association studies^6,7^ and further biomedical analyses^8^. In past studies, many phenotyping algorithms have been developed using EHRs^9–13^. Phenotyping is also expected to stratify patients into subgroups that reflect the molecular and clinical diversity of diseases^14^, which is expected to have substantial potential to contribute to risk estimation^15^, diagnostic support and therapeutic development^16^ and the determination of the best available care for each patient based on their stratified subgroups.

Prospective genome cohort studies are known as one of the most important resources for developing precision medicine^17,18^. In these cohort studies, a wide range of data, including lifestyle, genomic, molecular, medical record and phenotypic data^19–21^, are collected and analyzed to elucidate clinical problems. The Birth and Three-Generation Cohort Study (BirThree Cohort Study)^19^ of the Tohoku Medical Megabank (TMM) project is a large-scale prospective genome cohort study that recruited more than 70,000 subjects, including more than 20,000 pregnant women and their family members in 48 hospitals in Miyagi Prefecture from regional populations.

Hypertensive disorders of pregnancy (HDP) is one of the leading cause of maternal morbidity and mortality, affecting approximately 2–8%^22^ of all pregnancies, and is a clinical syndrome that mainly includes three subgroups: gestational hypertension (GH), preeclampsia (PE), and superimposed preeclampsia (SPE)^23^. It is known that there are major differences between the subgroups in the likelihood to develop severe complications and in pathogenesis^24–29^. However, accurately diagnosing these subgroups is quite difficult in clinical practice, and the same treatment has been applied across the several subgroups. In addition to the subgrouping, further classification into early onset (EO) and late onset (LO) based on the timing of onset has gained attention because of the different clinical forms with different pathophysiological features^30,31^. Because of these major differences in the subgroups of HDP, the precise classification of patient diseases into subgroups by phenotyping algorithms is necessary for genomic, epidemiologic and other biomedical analyses.

In hospital-based clinical research, clinical research coordinators (CRC) are able to identify patients precisely by creating a case report form (CRF) using clinical data, including clinical notes. However, there are severe limitations in the phenotyping of patients in large-scale cohort studies that lack of complete unstructured clinical notes and have limited accessible EHR data for partial subjects due to the lack of linkages in cohort data collection because the subjects of the cohort study are not patients of a hospital but are part of the regional population. Recently, the UK Biobank^32^ and All of Us^33^, which are large-scale prospective genome cohort studies, collected structured data on EHRs, such as diagnoses, laboratory results, medications, and billing codes, using record linkages; however, collection of clinical notes were still not achieved. Because of this limitation, it is not realistic to perform phenotyping of hundreds of thousands of subjects in a large-scale cohort using the conventional approach in clinical research. Consequently, it is necessary to develop new high-throughput and objective phenotyping algorithms that exploit clinical notes.

In this study, we developed two rule-based HDP phenotyping algorithms and programmatically implemented them that follow American and Japanese clinical guidelines because there are no internationally standardized guidelines for HDP, but domestic guidelines have been developed^34–37^. The developed algorithms were applied to phenotype approximately 23,000 pregnant women in the BirThree cohort. We also compared the phenotyped subgroups with a clinician chart review to evaluate the consistency of the clinical concepts between the developed algorithms and the clinical guidelines.

## Methods

### Development of hypertensive disorders of pregnancy (HDP) phenotyping algorithm 1

We developed HDP phenotyping algorithm 1 according to the American College of Obstetricians and Gynecologists (ACOG) guidelines^23^. Algorithm 1 detects HDP patients based on hypertensive disease history, blood pressure (BP), proteinuria (PU), timing of onset and PE-related clinical conditions (see “Details of the identification rules for the HDP subgroups in algorithm 1” section in the Supplemental Methods).

PU in early pregnancy is caused by several possibilities both related and unrelated to HDP, such as some degree of renal insufficiency and chronic kidney disease (CKD). To avoid overestimating the HDP group, we implemented rules to exclude subjects with PU in early pregnancy from the HDP group.

### Development of hypertensive disorders of pregnancy (HDP) phenotyping algorithm 2

We developed HDP phenotyping algorithm 2 according to the guidelines of the Japan Society of Obstetrics and Gynecology (JSOG) (see “Details of the identification rules for the HDP subgroups in algorithm 2” section in the Supplemental Methods). In algorithm 2, hypertensive subjects without PU were assigned to SPE or PE when the following conditions were met: maternal organ dysfunction (see Supplementary Table 1) or light-for-date. (see “Selection of subjects with light-for-date” section in the Supplemental Methods). The patients having maternal organ dysfunction were determined by analyzing clinical notes (see “Selection of subjects with maternal organ dysfunction” section in the Supplemental Methods). More details about the process used to reach the target conditions are provided in the “Details of the process used to analyze unstructured clinical notes” section in the Supplemental Methods.

### Implementation of the developed phenotyping algorithms

The developed phenotyping algorithms according to American and Japanese clinical guidelines were implemented by Python 3.8.10 and Perl 5.16.3 (https://github.com/ogishimalab/HDP_phenotyping_algorithms). The implementations take no arguments and take both data for phenotyping and the lists of subjects who have specific phenotype about exclusion and inclusion criteria of HDP and their subtypes. The lists were required to prepare before phenotyping by analysis of clinical notes performed by such as pattern matching based on rules (see “Details of the processes used to analyze unstructured clinical notes” and “Selection of subjects with light-for-date” in Supplemental Methods).

### Data sources used to apply the developed algorithms

We selected research subjects from approximately 23,000 pregnant women who were included in the BirThree Cohort study, which is a population-based prospective cohort study. We excluded subjects who did not have medical records at delivery, who did not have BP and PU data, who did not have a live birth and who withdrew from the study. Using these criteria, we included 22,452 research subjects in the dataset. The baseline characteristics of all pregnant women in the BirThree Cohort study are provided in a previous report^38^.

**Figure 1:**
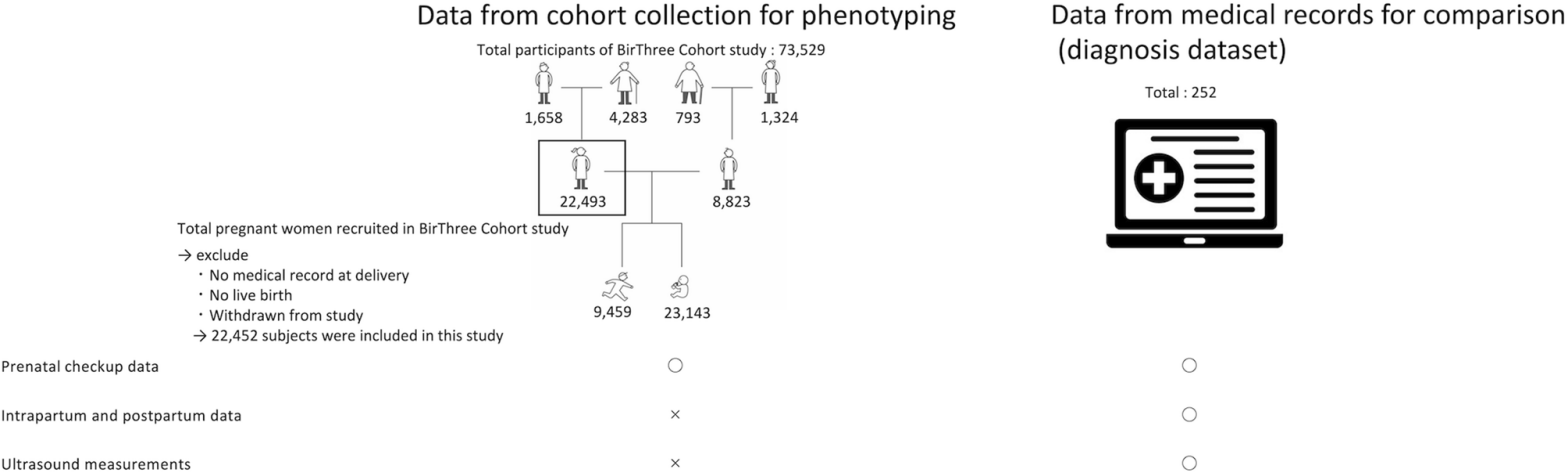
Overview of the sources of the cohort data collection and diagnosis dataset. The left side represents data from the cohort that consist of an interview conducted at the first visit, prenatal checkups and records at delivery. The right side represents the diagnosis dataset from the medical record used in validation. The diagnosis dataset consisted of both intrapartum and postpartum data and ultrasound measurements, in addition to cohort collection.

All data in our dataset were transcribed from the clinical records of usual care by trained genome medical research coordinators (GMRCs) from the interview conducted at the first visit, prenatal checkups and records at delivery (left side of Fig. 1). The average number of visits for prenatal checkups and measurements of BP and PU for pregnant women are shown in Supplementary Table 2. All items used in our phenotyping algorithms are listed in Supplementary Table 3.

### Comparison of phenotyped subgroups and diagnoses

We conducted a clinician chart review to compare the subgroups phenotyped by the algorithms with the diagnoses. The comparison was performed to evaluate the consistency of clinical concepts between the developed algorithms and the clinical guidelines. A clinician chart review was performed based on the American guidelines^23^ for 252 subjects who met the following criteria: (1) subjects recruited at Tohoku University Hospital between September 2015 and November 2016 and (2) subjects who were able to access their medical records. The clinician chart review was conducted by two obstetricians, a mid-career and a senior obstetrician. If the subgroups assigned by the two doctors differed, the subgroups were determined by discussion. The chart review was performed blindly and annotated in a binary manner for each subgroup of HDP, CH and normotensive. The baseline characteristics of the 252 subjects are shown in Supplementary Table 4.

We calculated positive predictive values (PPVs), negative predictive values (NPVs), accuracies, sensitivities and specificities to compare our algorithms against the diagnoses. To calculate sensitivity and specificity, we identified true positive (TP), true negative (TN), false positive (FP), false negative (FN) by comparison of identified subgroups between phenotyping algorithms and diagnosis of 252 subjects. Based on the four indices, we identified sensitivity and specificity as TP/(TP + FN) and TN/(TN + FP). We identified accuracy using scikit-learn in python3. The diagnosed subgroups of the 252 subjects are listed in Supplementary Table 5. In this study, there were differences in data coverage between the cohort data for phenotyping and the diagnosis dataset that only diagnosis dataset included both intrapartum and postpartum data and ultrasound measurements, in addition to cohort data (right side of Fig. 1). The all items used for the diagnoses are listed in Supplementary Table 6.

### Ethics declarations

This study was approved by the ethical committee of Tohoku Medical Megabank Organization, Tohoku University. We confirm that all methods took place in accordance with relevant guidelines and regulations, under informed consent from all participants.

## Results

### The developed HDP phenotyping algorithms

**Figure 2:**
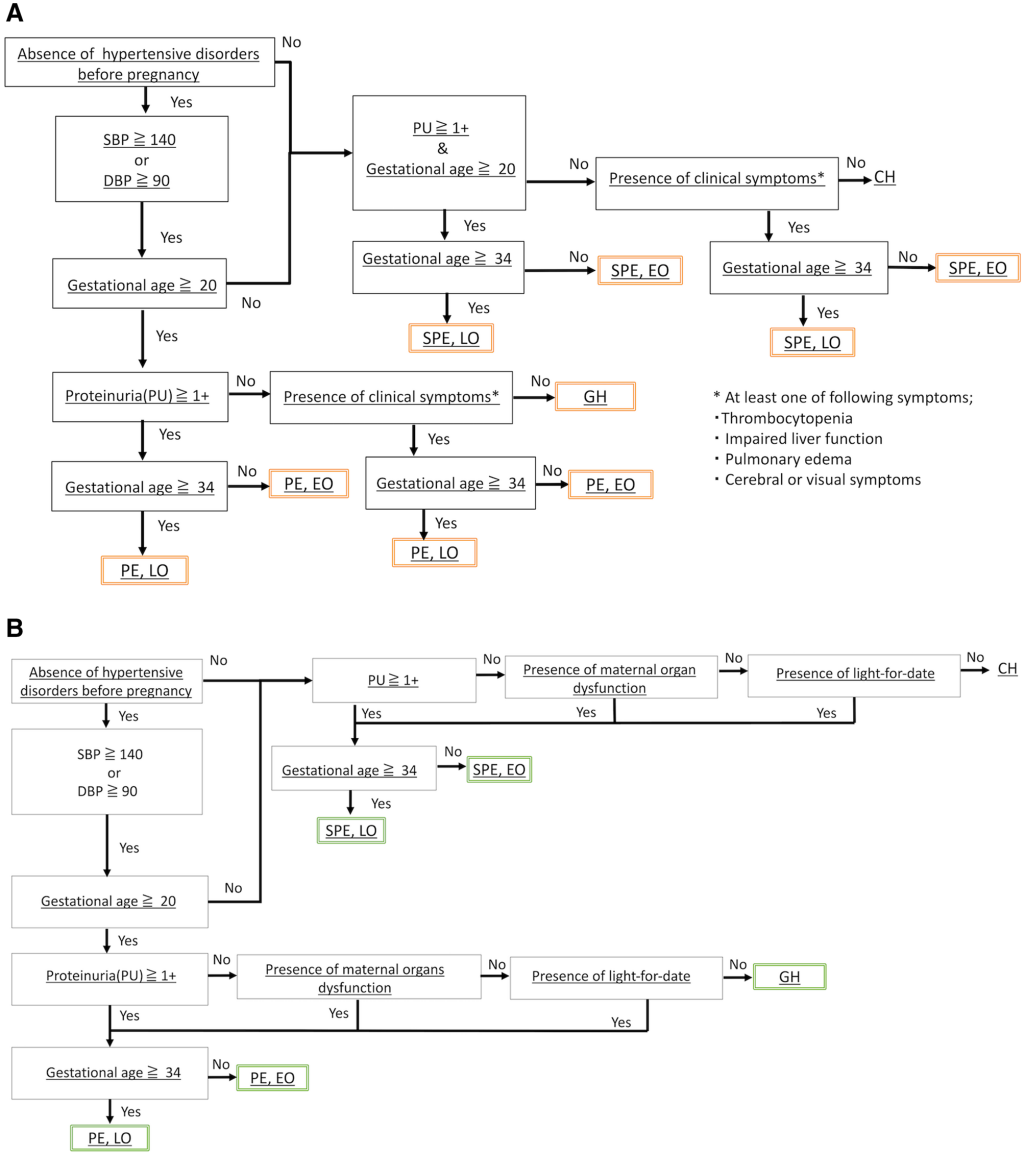
The developed rule-based phenotyping algorithms. (**a**) The rules of the developed HDP phenotyping algorithm 1. Phenotyping by algorithm 1 was conducted based on hypertensive disease history, BP, PU, timing of onset and PE-related conditions. The black boxes represent the conditions of rules, the arrow diagrams represent IF/THEN rules, and the orange boxes represent phenotyped subgroups. (**b**) The rules of the developed HDP phenotyping algorithm 2. The rules of the developed HDP phenotyping algorithm 2. Phenotyping by algorithm 2 was conducted with maternal organ dysfunction and light-for-date in addition to the factors included in algorithm 1.

The rules of the developed HDP phenotyping algorithms 1 and 2 are shown in Fig. 2a and b, respectively. The detailed results of the identification of subjects with a hypertensive disease history and maternal organ dysfunction by analyzing unstructured clinical notes are described in the “Details of the identified subjects with a hypertensive disease history and maternal organ dysfunction” section in the Supplemental Results. The detailed numbers of HDP subgroups phenotyped by the developed phenotyping algorithms are described in “The number of phenotyped subgroups of HDP” section.

### The number of phenotyped subgroups of HDP

Of the 22,452 research subjects, 1781 (7.93%) were phenotyped as having HDP by phenotyping algorithm 1. Among the subgroups of HDP, 890 subjects (3.96%) had GH, 304 (1.35%) had SPE, and 587 (2.61%) had PE. According to phenotyping with algorithm 2, 1813 (8.08%) subjects had HDP. Among the subgroups of HDP, 825 subjects (3.67%) had GH, 336 (1.50%) had SPE, and 652 (2.90%) had PE (Table 1). Compared with algorithm 1, fewer subjects had GH or CH, while more had PE or SPE according to algorithm 2 because the algorithm 2 classifies hypertensive subjects without PU into PE or SPE based on the criteria such as subjects having epigastralgia, HELLP syndrome, eclampsia and FGR, in addition to the criteria of algorithm 1.

**Table 1:** Number of subgroups phenotyped by the phenotyping algorithms.

	Algorithm 1		Algorithm 2	
	EO	LO	EO	LO
GH	356 (1.59%)	534 (2.38%)	325 (1.45%)	500 (2.23%)
SPE	207 (0.92%)	97 (0.43%)	219 (0.98%)	117 (0.52%)
PE	140 (0.62%)	447 (1.99%)	149 (0.66%)	503 (2.24%)
CH	542 (2.41%)		510 (2.27%)	
Normotensive	20,129 (89.65%)			
Total	22,452			

Among the subgroups phenotyped by algorithm 1 and 2, 97 (0.44%) subjects were classified into different subgroups of HDP (Supplementary Table 7).

The causes of the differences in the phenotyped HDP subgroups between algorithm 1 and 2 are shown in Supplementary Table 8. The sum of the subjects was not equal to 97 because one person may have had multiple symptoms and differences in the timing of the symptoms.

### Comparison of the subgroups identified by HDP phenotyping algorithms with diagnoses

Of the 252 subjects included in the diagnosis dataset, 36 (14.2%) were diagnosed as HDP. The subgroups of the 252 subjects are shown in Supplementary Table 5. The performance of phenotyping algorithm 1 and 2 compared to the diagnoses is shown in Tables 2 and 3, respectively. Comparisons for GH EO were not applicable because none of the 252 subjects was diagnosed with GH EO.

**Table 2:** The performance of phenotyping algorithm 1.

	Algorithm 1				
	PPV	NPV	Accuracy	Sensitivity	Specificity
HDP	0.96	0.96	0.96	0.72	1.00
GH	0.75	0.98	0.97	0.33	1.00
GH EO	–	–	–	–	–
GH LO	0.67	0.97	0.97	0.22	1.00
SPE	0.92	1.00	0.99	0.92	1.00
SPE EO	0.89	1.00	0.99	0.89	1.00
SPE LO	1.00	1.00	1.00	1.00	1.00
PE	0.91	0.98	0.98	0.67	1.00
PE EO	1.00	1.00	1.00	0.50	1.00
PE LO	0.90	0.98	1.00	0.50	1.00

**Table 3:** The performance of phenotyping algorithm 2.

	Algorithm 2				
	PPV	NPV	Accuracy	Sensitivity	Specificity
HDP	0.90	0.96	0.95	0.75	0.99
GH	0.67	0.97	0.97	0.22	1.00
GH EO	–	–	–	–	–
GH LO	0.50	0.97	0.96	0.11	1.00
SPE	0.80	0.99	0.99	1.00	0.99
SPE EO	0.82	1.00	0.99	1.00	0.99
SPE LO	0.75	1.00	1.00	1.00	1.00
PE	0.83	0.97	0.97	0.67	0.99
PE EO	1.00	1.00	1.00	0.50	1.00
PE LO	0.82	1.00	0.98	0.50	1.00

For phenotyping of HDP overall, the PPVs, NPVs, accuracies, and specificities of both algorithms 1 and 2 were high (0.96, 0.96, 0.96, and 1.00 for the PPVs, NPVs, accuracy and specificity of algorithm 1 and 0.90, 0.96, 0.95, and 0.99 for the PPV, NPV, accuracy and specificity of algorithm 2, respectively), and the sensitivities were modest (0.72 and 0.75 for algorithm 1 and 2, respectively). Of the 252 subjects in the diagnosis datasets, 15 and 16 subjects were classified into different subgroups by algorithm 1 and algorithm 2, respectively. Of the 15 subjects with different subgroups by algorithm 1, 10 of the differences were caused by missing intrapartum and postpartum data, 1 was caused by a missing creatinine level, 1 was caused by transcription error and 3 were caused by headache that was not accepted as an unexplained new-onset headache that was unresponsive in the chart review (see Supplementary Table 9).

Of the 16 subjects who showed differences by phenotyping algorithm 2, three were new because the subgroups phenotyped by algorithm 1 matched the diagnosed subgroups (see Supplementary Table 10).

## Discussion

In this study, we developed two HDP phenotyping algorithms, algorithm 1 and 2. Algorithm 1 identifies HDP patients based on the hypertensive disease history, BP, PU, timing of onset and PE-related clinical conditions. In addition to the factors included in algorithm 1, algorithm 2 conducts phenotyping by considering maternal organ dysfunction and light-for-date, which originate from placental dysfunction in early pregnancy^39^. In these phenotyping results, 97 (0.44%) subjects were phenotyped into different subgroups by the two algorithms, and they actually had clinical conditions related to maternal organ dysfunction, such as epigastralgia and light-for-date (Supplementary Table 8). The choice of which algorithm to use depends on whether the phenotyping will be performed with the clinical concepts of the American or Japanese clinical guidelines used in algorithm 1 and 2, respectively. The Japanese clinical guidelines of HDP have been repeatedly updated in the past^35,36,40,41^ and will be continually updated according to the progress of research to understand the pathogenesis of HDP. Therefore, algorithm 2 will be updated continuously according to updates to the Japanese guidelines.

In this study, the number of identified subjects with a hypertensive disease history (115; 0.51%) and underlying hepatic or renal disorders (323; 1.44%) by analysis of unstructured clinical notes was slightly smaller than that in previous reports^42,43^. This result suggests the possibility of the underestimation of subjects with these conditions because of differences between the diagnoses and the self-reported interviews, which were the sources of the transcribed data regarding disease history.

Of the 22,452 subjects in the BirThree cohort study, the proportions of the phenotyped HDP patients by algorithms 1 and 2 were almost identical to the previously reported prevalence rate of HDP^38,44,45^.

To evaluate the performances of our developed phenotyping algorithms, we performed chart review by two obstetricians for 252 among 22,452 research subjects (see “Comparison of phenotyped subgroups and diagnoses” section). Among 252 subjects, 203 (80.56%) were identified as normotensive, 13 (5.16%) were as CH, and 36 (14.29%) were as HDP patients. For HDP subgroups, we identified 9 subjects (3.57%) as GH, 15 (5.95%) as PE, and 12 (4.76%) as SPE (Supplementary Table 5).

The prevalence of HDP in the diagnosis dataset was 14.29%, which was higher than that in the full cohort (Supplementary Table 5). The baseline characteristics of the diagnosis dataset were higher risk of HDP in terms such as BMI and age than the full cohort (Supplementary Table 4). These differences may have occurred because the subjects in the diagnosis dataset were recruited at Tohoku University Hospital, which is a leading hospital that actively accepts high-risk cases, including patients with HDP.

In the evaluation of the performances of our developed algorithms, our phenotyping algorithms showed high PPVs, NPVs, accuracies and specificities and modest sensitivities, despite the limitation of data availability of intrapartum and postpartum data (Table 2, 3). In the results comparison between algorithm 1 and diagnosis, 15 of the 252 subjects in the diagnosis dataset were phenotyped into different subgroups of HDP by algorithm 1 (Supplementary Table 9). All of these 15 differences will be resolved by linking EHRs and cohort data^19^ because all differences were caused by lacking intrapartum and postpartum data and detailed description about headaches in cohort data. In the comparison for algorithm 2, 16 of 252 subjects were phenotyped into different subgroups of HDP (Supplementary Table 10). In particular, three of the sixteen differences are caused by fit to criteria of algorithm 2 added to algorithm 1, and these are not included in the diagnostic criteria. These results show that the major cause of miss-phenotyping was not inadequacy of phenotyping algorithms but differences of data sources between phenotyping and diagnosis. Based on these results, we concluded that there was consistency in clinical concepts between phenotyping algorithms and clinical guidelines. Furthermore, these results showed that our phenotyping algorithms are ready to be applied in HDP phenotyping tasks.

We used the transcribed medical records of the BirThree Cohort study as data sources for the phenotyping algorithms. The advantage of using the BirThree Cohort study data is the high representativeness of over 70% of the pregnant women in the regional population during the study period. This study is premised on the representativeness of the data sources because we evaluated the performances of our algorithms by comparing the number of subjects having HDP between the study population and the prevalence of HDP in previous reports, and this was large enough to compare the proportion of phenotyped subgroups of HDP with the prevalence rates.

## Conclusions

We developed two phenotyping algorithms to identify subjects with HDP and their subtypes from large-scale cohort data collection to perform further biomedical analyses. Our algorithms were developed according to American and Japanese guidelines that differ in the criteria for HDP and its subtypes. Compared to the diagnosis, our phenotyping algorithms showed high PPVs (0.96 and 0.90 for algorithm 1 and 2, respectively), NPVs (0.96 for algorithms 1 and 2), accuracies (0.96 and 0.95 for algorithm 1 and 2, respectively) and specificities (1.00 and 0.99 for algorithm 1 and 2, respectively), and modest sensitivities (0.72 and 0.75 for algorithm 1 and 2, respectively), despite the limitation of data availability for intrapartum and postpartum data in the cohort collection. The application of our phenotyping algorithms to large-scale cohort data will substantially accelerate HDP research by facilitating the high-throughput and precise identification of patients with HDP.

### Supplementary Information

Supplementary Information 1.

Supplementary Tables.

## Data Availability

The data underlying this article were provided by the Tohoku Medical Megabank project with permission. Data will be shared on request to the Tohoku Medical Megabank project with permission.
