# Supplementary material for: Development of phenotyping algorithms for hypertensive disorders of pregnancy (HDP) and their application in more than 22,000 pregnant women
Source: Sci Rep. 2024 Mar 15;14:6292. doi: 10.1038/s41598-024-55914-9 (PMC10943000; doi:10.1038/s41598-024-55914-9)
Supplement: Supplementary file 1 — Supplementary Information 1. [file 41598_2024_55914_MOESM1_ESM.pdf]

SUPPLEMENTAL METHODS

Details of the identification rules for the HDP subgroups in algorithm 1

In algorithm 1, the subgroups of HDP were defined as follows using blood pressure and urine dipstick tests at each prenatal checkup. In algorithm 1, hypertension was defined as a systolic blood pressure (BP) > 140 mm, diastolic BP > 90 mm or both at one prenatal checkup and the same condition in a remeasurement during the same visit. GH was defined as a lack of chronic hypertension and presentation with hypertension after 20 gestational weeks in the absence of proteinuria. PE was defined under the following conditions: a lack of chronic hypertension, a lack of proteinuria in early pregnancy, the presence of hypertension, and proteinuria ≥ 1+ after 20 gestational weeks; PE can also occur if at least one of the PE-related conditions were met with a lack of proteinuria after 20 gestational weeks. SPE was defined as hypertension before 20 gestational weeks complicated with proteinuria or at least one of PE-related conditions were met after 20 gestational weeks. We used the following as PE-related conditions: PLT<100,000/μL, GOT≧70 international units/L (IU), GPT≧70 IU, pulmonary edema, headaches after hypertension and phosphene. CKD in early pregnancy was defined as PU ≥ 1+ before 20 gestational weeks.

The timing of onset for each subgroup was defined by the gestational age when the following conditions were met. The onset of GH was determined by the timing of hypertension onset. PE onset was determined by the timing of the confirmation of both hypertension and proteinuria. The onset of SPE was determined by the following two criteria: 1) the timing of confirmation of proteinuria or PE-related conditions in subjects with hypertensive disorders pre-

pregnancy and 2) the timing of confirmation of both hypertension and proteinuria or PE-related conditions in subjects with hypertension before 20 gestational weeks.

We defined the classification of the timing of onset as follows: early-onset preeclampsia (EO-PE) occurred before or at 33 gestational weeks, and late-onset preeclampsia (LO-PE) occurred at 34 gestational weeks or later. In our algorithm, we defined hypertensive patients as those with a blood pressure at least one point over the cutoff for hypertension during a prenatal checkup. We used the last measurement of blood pressure on the day that the blood pressure values were measured.

Details of the identification rules for the HDP subgroups in algorithm 2

The timing of the onset of PE was determined based on the timing of the observation of both hypertension and maternal organ dysfunction. The timing of the onset of SPE was determined based on the timing of the observation of maternal organ dysfunction in subjects who had hypertension before 20 gestational weeks and the timing of the observation of both hypertension and maternal organ dysfunction in subjects who had hypertensive disorders before pregnancy.

Identification of subjects with a hypertensive disease history

We categorized subjects who met one of the following criteria as having hypertensive disorders before pregnancy: 1) pregnant women with hypertension as an underlying disease, 2) pregnant women who received antihypertensive treatments before pregnancy, and 3) pregnant women who visited the Department of Endocrinology or Nephrology. Antihypertensive medications found in medical records are listed in Supplementary Table 11.

The third condition was set to select pregnant women who received antihypertensive treatments because of nephrological diseases such as primary aldosteronism.

Selection of subjects with maternal organ dysfunction

We selected subjects who met one of the following criteria as having maternal organ dysfunction: 1) pregnant women with newly diagnosed hepatic or renal disorders, 2) pregnant women with neurological dysfunctions, and 3) pregnant women with hematological dysfunctions. The detailed list of conditions of maternal organ dysfunction and the number of subjects who met each condition are summarized in Supplementary Table 1. The list of conditions of maternal organ dysfunction was defined according to expert knowledge. In the second condition, we regarded only headaches of hypertensive patients as neurological dysfunction-related headaches because almost all headaches of normotensive subjects were caused by factors other than HDP, such as common colds and migraines. Other conditions of maternal organ dysfunction, including high creatinine levels and abnormal umbilical artery Doppler waveforms, were not included in our algorithm because of missing data.

Details of the processes used to analyze unstructured clinical notes

We extracted terms related to conditions and medications about hypertensive disorders before pregnancy, underlying hepatic or renal disorders and maternal organ dysfunction. Based on the obtained terms, we identified subjects who had extracted term-related conditions and subjects who received antihypertensives before pregnancy from unstructured clinical notes from the prenatal checkup and first-visit interview data. The input text data included the disease history, major complaints, treatments, and medications prescribed at each prenatal checkup. Our

approach consisted of the following four steps: A) preprocessing of raw clinical notes, B) extracting terms related to the target conditions and treatments from preprocessed clinical notes, C) extracting patterns of denial and already cured underlying diseases, and D) identifying target subjects using partial matching of extracted words and denial patterns to sentences of clinical notes. Step A, which is preprocessing, was performed as follows: 1) we splitted clinical notes from the first-visit interview and prenatal checkup into sentences by separator characters, and 2) we performed morphological analysis using Mecab [1] with Manbyo-Dictionary [2] as the corpus. The outputs of preprocessing were sentences and contained words with the part-of-speech for all words and ICD10 codes for disease names. Step B was performed to extract both words related to the target conditions, including underlying hepatic or renal disorders and maternal organ dysfunction, and words related to the medications of hypertension. We extracted words related to the target condition by manual curation from words with ICD10 codes. To extract words related to antihypertensives, we extracted all nouns obtained in the preprocessing step, excluded unnecessary words (e.g., words consisting of only kanji because medications never contain kanji) and obtained words to represent antihypertensives manually by referring to the KEGG drug database and package labeling inserts for the remaining words. The terms obtained in step B were reviewed by two physicians to obtain suitable terms for each condition. The finalized list of terms is provided in Supplementary Tables 1, 11 and 13. Step C, which is the extraction of patterns of denial and already-cured underlying diseases, was performed by manual curation from sentences containing extracted terms in step B. The obtained denial patterns are provided in Supplementary Table 12. Finally, step D was performed to identify subjects with target conditions and subjects who received antihypertensives. Step D was performed as follows: 1) partial matching of the words obtained in process B to sentences

obtained in step A was performed using the regular expression of Perl functions to extract sentences containing target words, and 2) the removal of sentences with denial patterns obtained in step C was performed using partial matching.

Selection of subjects with light-for-date

We selected subjects who met the criterion of having less than -1.5 times the birth weight standard deviation score (SDS) of newborn as those with light-for-date. The SDS of birthweight was computed with the Excel-based computational tool of the Japanese Society of Pediatric Endocrinology and the Japanese Society for Human Auxology (http://jspe.umin.jp/medical/files_chart/taikakubirthlongcross_v1.1.xlsx (last access: Apr 4, 2019)) according to the Japanese fetal growth curve [3].

SUPPLEMENTAL RESULTS

Details of the identified subjects with a hypertensive disease history and maternal organ dysfunction

In the results analysis of unstructured clinical notes, 115 (0.51%) and 445 (1.98%) subjects were identified as subjects with a hypertensive disease history and maternal organ dysfunction, respectively. Of the 115 subjects with a hypertensive disease history, 113 (0.50%) subjects had hypertension as an underlying disease, 7 (0.03%) subjects received hypertensive treatment, and 3 (0.01%) subjects visited the Department of Endocrinology or Nephrology.

Of the 445 subjects with maternal organ dysfunction, 194 (0.86%) subjects had newly diagnosed hepatic or renal disorders, 177 (0.79%) subjects had neurological dysfunction, and

103 (0.46%) subjects had hematologic dysfunction. In the analysis of the unstructured clinical notes, 323 (1.44%) subjects were identified as having underlying hepatic or renal disorders, excluding them from the analysis of subjects with newly hepatic or renal disorders. The terms most commonly used to describe underlying hepatic or renal disorders in our analysis were nephritis and hepatitis (109 and 75 subjects, respectively). The terminology and number of cases of underlying hepatic and renal disorders are shown in Supplementary Table 13. The most common condition among maternal organ dysfunctions was headaches after hypertension (148 subjects). The terminology, conditions and number of cases of maternal organ dysfunction are shown in Supplementary Table 1.

Identification of subjects with light-for-date

Of the 22,452 research subjects, 152 were not included in the calculation of the SDS because of missing data. Of the remaining 22,300 subjects, 986 (4.42%) were identified as having light-for-date. Among the 986 subjects with light-for-date, 178 experienced hypertension during pregnancy, and 33 had maternal organ dysfunction.

REFERENCES

[1] T. Kudo, K. Yamamoto, Y. Matsumoto, Applying conditional random fields to Japanese morphological analysis, in: Proceedings of the 2004 Conference on Empirical Methods in Natural Language Processing, ACL, Barcelona, Spain, 2004, pp. 230–237.

[2] MANBYO Dictionary, Ikoma: social computing laboratory, NAIST. http://sociocom.jp/~data/2018-manbyo/index.html, 2019 (accessed 5 March 2020).

[3] K. Itabashi, M. Fujimura, S. Kusuda, M. Tamura, T. Hayashi, T. Takahashi, Introduction of new gestational age-specific standards for birth size, J. Jpn. Pediatr. Soc. 114 (2010) 1271–1293.

[4] K. Watanabe, K. Matsubara, O. Nakamoto, J. Ushijima, A. Ohkuchi, K. Koide, S. Makino, K. Mimura, M. Morikawa, K. Naruse, K. Tanaka, T. Nohira, H. Metoki, I. Kawabata, S. Takeda, H. Seki, K. Takagi, M. Yamasaki, A. Ichihara, T. Kimura, S. Saito, Outline of the new definition and classification of "Hypertensive Disorders of Pregnancy (HDP)"; a revised JSSHP statement of 2005, Hypertens. Res. Pregnancy 6 (2018) 33–37. https://doi.org/10.14390/jsshp.HRP2018-014.
