# Supplementary material for: Development of phenotyping algorithms for hypertensive disorders of pregnancy (HDP) and their application in more than 22,000 pregnant women
Source: Sci Rep. 2024 Mar 15;14:6292. doi: 10.1038/s41598-024-55914-9 (PMC10943000; doi:10.1038/s41598-024-55914-9)
Supplement: Supplementary file 2 — Supplementary Tables. [file 41598_2024_55914_MOESM2_ESM.pdf]

Supplementary Table 1. Terminology and conditions categorized as maternal organ dysfunction and the number of patients with each condition.

Maternal organ dysfunction-related condition	N (%)
Headaches after hypertension	148 (0.66)
Hepatic dysfunction	31 (0.14)
Epigastralgia	29 (0.13)
Renal dysfunction	16 (0.07)
Phosphene	18 (0.08)
HELLP syndrome	18 (0.08)
GOT≧70 international units/L(IU)	41 (0.18)
GPT≧70 IU	75 (0.33)
PLT<100,000/μL	97 (0.42)
Visited the Department of Endocrinology or Nephrology	9 (0.04)
Pulmonary edema	6 (0.03)
Eclampsia	6 (0.03)
Eclamptic seizures	4 (0.0)
Nephrotic syndrome	3 (0.01)
Liver enzyme elevation	4 (0.02)
Tunnel vision	5 (0.02)

Supplementary Table 2. The average number of visits for prenatal checkups and the average number of measurements of blood pressure and proteinuria for pregnant women

Item	Mean (SD)
The average number of visits that pregnant women attended for prenatal checkups	16.8 (3.4)
The average number of measurements of blood pressure	12.3 (3.2)
The average number of measurements of proteinuria	12.0 (2.8)

Supplementary Table 3. Data items used in phenotyping algorithms 1 and 2.

Item
Systolic blood pressure
Diastolic blood pressure
Proteinuria
Gestational age at each prenatal checkup
Hypertensive disorders before pregnancy
Maternal organ dysfunction
Light-for-date

Supplemental Table 4. The baseline characteristics of the reference standard.

Variable	n	Age at enrollment, years					
		Total %	≦24 %	25-29 %	30-34 %	35-39 %	≧ 40 %
Number	252		10	56	93	69	24
Parity	250						
0		46.8	80	53.6	44.1	38.2	52.2
1		34.0	10.0	35.7	36.6	35.3	26.1
≧2		19.2	10.0	10.7	19.35	26.5	21.7
Pre-pregnancy BMI, kg/m^2	242						
<18.5		13.6	12.5	24.1	10.0	13.2	4.5
18.5 to <25		67.9	87.5	64.8	65.6	73.5	63.6
25 to <30		9.1	0.0	3.7	11.1	8.8	13.6
≧30		9.5	0.0	7.4	13.3	4.4	18.2
Smoking status	245						
Never smoked		65.6	50.0	70.4	75.0	51.5	60.9
Past smoker before pregnancy		25.8	12.5	22.2	15.2	41.2	34.8
Past smoker after pregnancy		7.0	37.5	7.4	7.6	4.4	4.3
Current smoker		1.6	0.0	0.0	2.2	2.9	0.0
Alcohol consumption	245						
Current drinker		18.8	12.5	22.2	17.4	19.1	13.8
Past drinker		36.7	50.0	38.9	30.4	35.3	44.8
Never drinks		39.6	37.5	37.0	46.7	36.8	20.7
Constitutionally never drinks		4.9	0.0	1.85	5.4	8.8	0.0
Fertility treatment	244						
Natural pregnancy		83.3	100	92.5	83.7	76.9	72.7
AIH		4.3	0.0	3.8	4.7	3.1	9.1
IVF		1.3	0.0	0.0	0.0	3.1	4.5
ICSI		7.3	0.0	1.9	8.1	10.8	9.1
Other		3.8	0.0	1.9	3.5	6.2	4.5
Education background	173						
Elementary/junior high school		2.9	11.1	5.1	1.6	1.9	0.0
High school		27.2	33.3	43.6	18.0	22.6	36.4

Vocational college		21.4	22.2	20.5	24.6	18.9	18.2
Junior College and Technical College		6.4	11.1	2.6	1.6	13.2	9.1
University		35.8	11.1	25.6	50.8	34.0	18.2
Graduated School		5.8	0.0	2.6	3.3	9.4	18.2
Other		0.6	11.1	0.0	0.0	0.0	0.0
Occupation	244						
Housewife or unemployed		36.1	37.5	33.3	35.9	41.2	27.2
Employed		63.9	62.5	66.7	64.1	58.8	72.7
Student		0.0	0.0	0.0	0.0	0.0	0.0
Household income, million Japanese yen/year	240						
<2		6.3	22.2	12.0	4.4	4.5	0.0
2 to <4		24.8	55.6	30.0	27.5	11.9	28.6
4 to <6		30.7	11.1	28.0	27.5	35.8	42.9
6 to <8		19.7	0.0	20.0	22.0	22.4	9.5
8 to <10		6.7	11.1	10.0	4.4	7.5	4.8
≧10		11.8	0.0	0.0	14.3	17.9	14.3

Supplemental Table 5. The number of subjects within the subgroups for the reference standard, algorithm 1 and algorithm 2.

Subgroups of the reference standard	N (%)	Subgroups of hypertensive subjects in Algorithm 1 (N, %)	Subgroups of hypertensive subjects in Algorithm 2 (N, %)
GH EO	0 (0.0)	356 (1.59)	325 (1.45)
GH LO	9 (3.57)	534 (2.38)	500 (2.23)
SPE EO	9 (3.57)	207 (0.92)	219 (0.98)
SPE LO	3 (1.19)	97 (0.43)	117 (0.52)
PE EO	3 (1.19)	140 (0.62)	149 (0.66)
PE LO	12 (4.76)	447 (1.99)	503 (2.24)
CH	13 (5.16)	542 (2.41)	510 (2.27)
Normotensive	203 (80.56)	20,129 (89.65)	20,129 (89.65)
Total	252	22,452	22,452

Supplementary Table 6. Data items used in the clinician chart review.

Item
Systolic blood pressure
Diastolic blood pressure
Result of urine dipstick tests
Gestational age at data collection
Hypertensive disorders before pregnancy
PLT at delivery
GOT at delivery
GPT at delivery
Urine protein-to-creatinine ratio
Impairment of hepatic function
Impairment of renal function
HELLP syndrome
Presence of eclamptic seizures
Disease history

Supplementary Table 7. The number of phenotyping differences between algorithm 1 and 2

Algorithm 1	Algorithm 2	N
GH EO	PE EO	9
	PE LO	22
CH	SPE EO	12
	SPE LO	20
GH LO	PE LO	34
PE LO	PE EO	14
SP LO	SP EO	37
Total		97

Supplementary Table 8. The causes of the differences in phenotyped HDP subgroups between algorithm 1 and 2

Symptoms	N
Light-for-date	85
Epigastralgia	3
Hepatic dysfunction	3
HELLP syndrome	2
Nephrotic syndrome	2
Eclampsia	1
Renal dysfunction	1
Visited the department of endocrinology or nephrology	1

Supplementary Table 9. The detailed cause of the differences between the HDP subgroups phenotyped by algorithm 1 and the diagnoses

Diagnosed subgroup	Phenotyped subgroup	*N*	Cause of difference
PE LO	CH	1	Missing creatinine level
PE LO	Normotensive	2	Missing intrapartum and postpartum data
SPE EO	CH	1	Missing intrapartum and postpartum data
GH LO	Normotensive	5	Missing intrapartum and postpartum data
PE EO	Normotensive	1	Missing intrapartum and postpartum data
PE LO	GH LO	1	Missing intrapartum and postpartum data
GH LO	GH EO	1	Transcription error
CH	SPE LO	1	The patient's headache was not accepted as an unexplained new-onset headache that was unresponsive in the chart review
CH	SPE EO	1	The patient's headache was not accepted as an unexplained new-onset headache that was unresponsive in the chart review
GH LO	PE LO	1	The patient's headache was not accepted as an unexplained new-onset headache that was unresponsive in the chart review

Supplementary Table 10. The detailed cause of the differences between the HDP subgroups phenotyped by algorithm 2 and the diagnoses

Diagnosed subgroup	Phenotyped subgroup	*N*	Cause of difference
PE LO	CH	1	Missing creatinine level
PE LO	Normotensive	2	Missing intrapartum and postpartum data
GH LO	Normotensive	5	Missing intrapartum and postpartum data
PE EO	Normotensive	1	Missing intrapartum and postpartum data
PE LO	GH LO	1	Missing intrapartum and postpartum data
GH LO	GH EO	1	Transcription error
CH	SPE EO	1	Maternal organ dysfunction
CH	SPE EO	1	Light-for-date
CH	SPE LO	1	Maternal organ dysfunction
GH LO	PE LO	1	Light-for-date
GH LO	PE LO	1	Maternal organ dysfunction

Supplementary Table 11. The list of antihypertensive medications used to identify subjects with hypertensive disorders before pregnancy.

Antihypertensive medications
Nifedipine
Amlodipine besylate
Olmesartan medoxomil
Methyldopa hydrate
Labetalol hydrochloride
Indapamide
Hydralazine hydrochloride
Aldosterone antagonist

Supplemental Table 12. The list of data items used to select subjects with underlying hepatic or renal disorders and the number of patients with each disorder.

Symptom	N (%)
Nephritis	109 (0.49)
Hepatitis	75 (0.33)
Pyelonephritis	70 (0.31)
IgA nephropathy	34 (0.15)
Pyelitis	36 (0.16)
Acute hepatitis	23 (0.10)
Hepatic dysfunction	13 (0.06)
Renal calculus	11 (0.05)
Hepatitis B	19 (0.08)
Fatty liver	6 (0.03)
Hydronephrosis	6 (0.03)
Acute nephritis	5 (0.02)
Liver cirrhosis	5 (0.02)
Primary biliary cirrhosis	4 (0.02)
Chronic glomerulonephritis	4 (0.02)
Renal aplasia	4 (0.02)
Hepatic hemangioma	3 (0.01)
Renal dysfunction	3 (0.01)
Renal failure	2 (0.01)
Renal disease	1 (0.00)
Drug-induced hepatitis	2 (0.01)
Renal glycosuria	2 (0.01)
Fulminant hepatitis	2 (0.01)
Medullary cystic kidney disease	1 (0.00)
Solitary kidney	1 (0.00)
Floating kidney	4 (0.02)
Liver enzyme elevation	1 (0.00)
Hepatolithiasis	1 (0.00)
Hepatic adhesions	1 (0.00)

Renal infarction	1 (0.00)
Pyeloureteral junction stenosis	1 (0.00)
Renal tumor	1 (0.00)
Lupus nephritis	1 (0.00)
Polycystic kidney	1 (0.00)
Renal hypoplasia	1 (0.00)
Solitary kidney	1 (0.00)
Cytomegalovirus hepatitis	1 (0.00)
Hereditary nephritis	1 (0.00)
Hypertensive nephrosclerosis	1 (0.00)
Membranoproliferative glomerulonephritis	1 (0.00)
Kidney rupture	1 (0.00)
Decreased liver function	1 (0.00)
Malignant renal tumor	1 (0.01)
Alcoholic cirrhosis	1 (0.01)
EB viral hepatic dysfunction	1 (0.01)
Hepatitis C	11 (0.04)
Autoimmune hepatitis	3 (0.01)
Membranous nephropathy	1 (0.00)
Purpura nephritis	1 (0.00)
Congenital horseshoe kidney	1 (0.00)

Supplemental Table 13. The denial patterns for obtaining treatments and clinical conditions

taking for a short period of time.
not on medication
medication in the past
without
dosed for prevention
careful and cautious follow-up
from about junior high school
minus
no seizures
(-)
leg seizures
hard to conceive
negative
headache caused by a cold
headache caused by fever
migraine headache
habitual headache
headache with sore throat
suffering from headaches before pregnancy
seizures of round ligament
seizure-like motions
already cured
suspicion
carrier
discontinuation of administration
complete recovery
